# Quantifying Cancer Absolute Risk and Cancer Mortality in the Presence of Competing Events after a Myotonic Dystrophy Diagnosis

**DOI:** 10.1371/journal.pone.0079851

**Published:** 2013-11-13

**Authors:** Shahinaz M. Gadalla, Ruth M. Pfeiffer, Sigurdur Y. Kristinsson, Magnus Björkholm, James E. Hilbert, Richard T. Moxley, Ola Landgren, Mark H. Greene

**Affiliations:** 1 Clinical Genetics Branch, Division of Cancer Epidemiology and Genetics, National Cancer Institute, National Institutes of Health, Bethesda, Maryland, United States of America; 2 Biostatistics Branch, Division of Cancer Epidemiology and Genetics, National Cancer Institute, National Institutes of Health, Bethesda, Maryland, United States of America; 3 Department of Medicine, Division of Hematology, Karolinska University Hospital Solna and Karolinska Institutet, Stockholm, Sweden; 4 Faculty of Medicine, University of Iceland and Department of Hematology, Landspitali National University Hospital, Reykjavik, Iceland; 5 Department of Neurology, Neuromuscular Disease Center, University of Rochester Medical Center, Rochester, New York, United States of America; 6 Metabolism Branch, National Cancer Institute, National Institutes of Health, Bethesda, Maryland, United States of America; University of Texas MD Anderson Cancer Center, United States of America

## Abstract

Recent studies show that patients with myotonic dystrophy (DM) have an increased risk of specific malignancies, but estimates of absolute cancer risk accounting for competing events are lacking. Using the Swedish Patient Registry, we identified 1,081 patients with an inpatient and/or outpatient diagnosis of DM between 1987 and 2007. Date and cause of death and date of cancer diagnosis were extracted from the Swedish Cause of Death and Cancer Registries. We calculated non-parametric estimates of absolute cancer risk and cancer mortality accounting for the high non-cancer competing mortality associated with DM. Absolute cancer risk after DM diagnosis was 1.6% (95% CI=0.4-4%), 5% (95% CI=3-9%) and 9% (95% CI=6-13%) at ages 40, 50 and 60 years, respectively. Females had a higher absolute risk of all cancers combined than males: 9% (95% CI=4-14), and 13% (95% CI=9-20) *vs.* 2% (95%CI= 0.7-6) and 4% (95%CI=2-8) by ages 50 and 60 years, respectively) and developed cancer at younger ages (median age =51 years, range=22-74 *vs.* 57, range=43-84, respectively, p=0.02). Cancer deaths accounted for 10% of all deaths, with an absolute cancer mortality risk of 2% (95%CI=1-4.5%), 4% (95%CI=2-6%), and 6% (95%CI=4-9%) by ages 50, 60, and 70 years, respectively. No gender difference in cancer-specific mortality was observed (p=0.6). In conclusion, cancer significantly contributes to morbidity and mortality in DM patients, even after accounting for high competing DM mortality from non-neoplastic causes. It is important to apply population-appropriate, validated cancer screening strategies in DM patients.

## Introduction

Myotonic Dystrophy (DM) is the most common adult muscle dystrophy. It is a chronic, slowly-progressive, autosomal dominant, multisystem disorder. Two major DM types have been identified: type 1 (DM1), caused by a CTG repeat expansion in the 3’ untranslated region of the dystrophia myotonica-protein kinase (DMPK) gene (chromosome 19q13.2)[1–3], and type 2 (DM2), caused by a CCTG repeat expansion in intron 1 of the zinc finger 9 (*ZNF9*) gene (chromosome 3q21)[4,5]. In addition to progressive skeletal muscle weakness and myotonia, DM patients may suffer from cardiac conduction defects, insulin-resistance, testicular atrophy, and respiratory insufficiency [6]. The complex clinical phenotype of this disease has been attributed to dysregulation in the pre-mRNA splicing process of multiple genes [4,7–9]. 

Several prior studies have suggested a high risk of premature death in DM patients (mean age at death: early 50s) [10–13]. The main reported causes of death were respiratory and cardiac complications, followed by malignancy. Nonetheless, until recently, DM patients were not formally considered to be at a clinically significant increased risk of cancer. 

Using the Swedish and Danish population-based registries, we previously reported that patients with DM (n=1,658) were at high risk of cancers of the brain, colon, endometrium, ovary, and possibly cancers of the eye, thyroid gland, and non-melanoma skin [14]. The excess risks of thyroid and eye cancers have been replicated in a subsequent clinic-based study of 307 DM cases [15]. While the comparison of risk among DM patients in relation to the general population provides etiological insights, the absolute risk estimates (also called cumulative incidence) are more useful for the clinical management of individual patients. 

Therefore, in the current study, we used the Swedish population-based registries to provide a comprehensive assessment of the absolute risk of cancer incidence and cancer mortality after DM diagnosis, while statistically accounting for the high competing risks from non-cancer deaths among patients with this disorder. 

## Subjects and Methods

### Swedish Population Registries and Study Participants

From the Swedish Patient Registry, we identified all individuals with a DM discharge diagnosis (the International Classification of Diseases (ICD) 9^th^ version=359C or ICD-10=G711) between January 1, 1987 and December 31, 2007 (n=1,121). The Swedish Patient Registry began in 1964 and reached 100% coverage of nationwide hospitalizations in 1987. Outpatient data were added starting in 2000. All Registry diagnoses were coded according to either ICD-9: 1987-1995, or ICD-10, thereafter [16,17].

**Figure 1:**
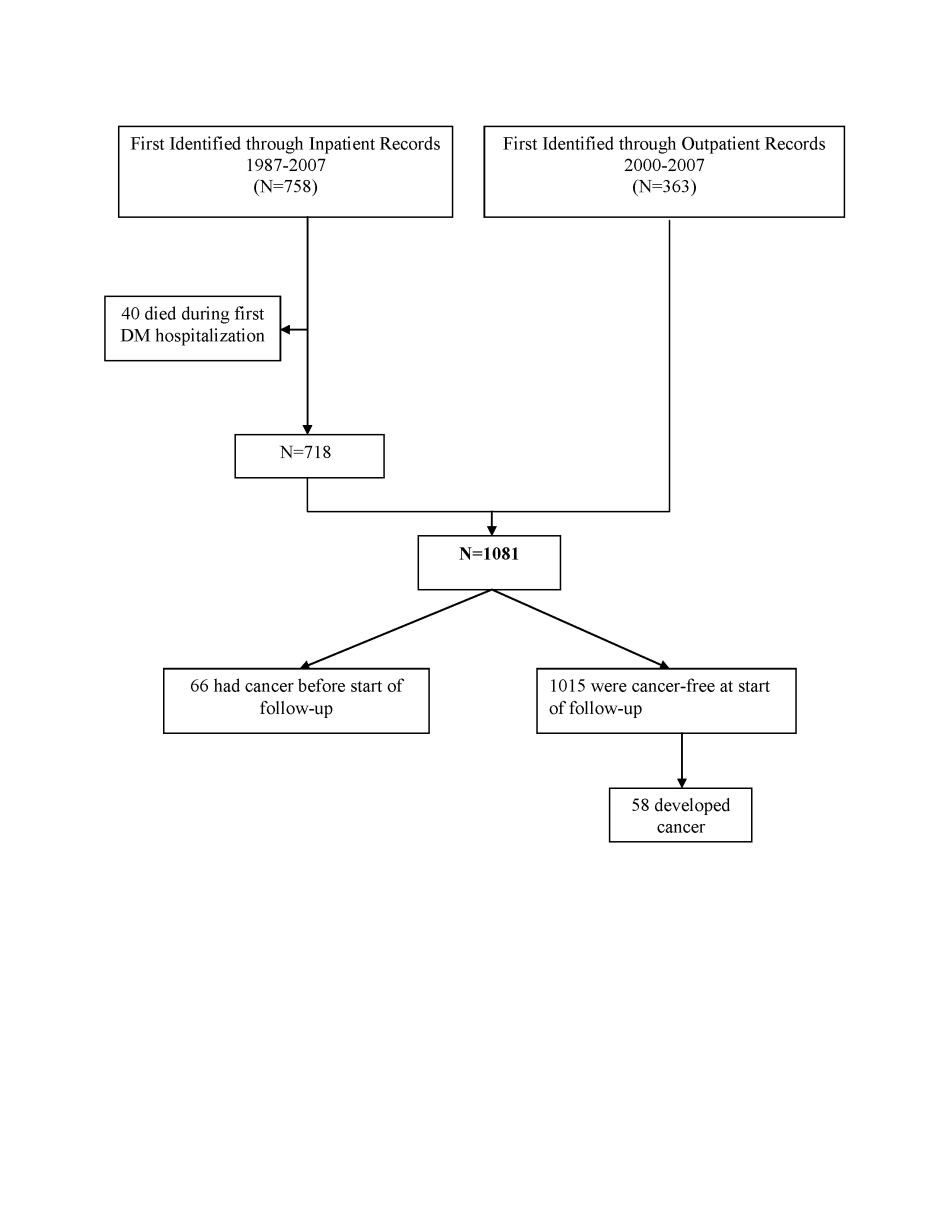
Flow chart describing the selection of study participants from the Swedish Patient Registry.

Using each individual’s national identification number, DM patients were linked to the Swedish Cause of Death Registry to obtain date and cause of death. Forty DM subjects were excluded because they died during their first DM hospitalization. The remaining 1,081 DM patients were linked to the Swedish Cancer Registry to ascertain cancer diagnosis. The Swedish Cancer registry has collected detailed information on incident cancer cases throughout Sweden since 1958. All cancer diagnoses were coded according to the ICD-7 through ICD-10. The completeness and diagnostic accuracy of the cancer registry exceeded 95% in validation studies [18,19]. When calculating absolute risk of cancer after a DM diagnosis, we included only DM patients who had not been diagnosed with cancer prior to the date of first DM diagnosis (n=1,015). Only first primary cancers were included in this analysis. Death and cancer data were available through December 31, 2007. Figure 1 summarizes patient selection for this study. 

### Ethics Statement

The study was performed in accordance with the ethical standards laid down in the 1964 Declaration of Helsinki. The requirement for informed consent was waived because we had neither direct contact with, nor personal identifying information from, study subjects. An exemption from the National Institute of Health (NIH) Institutional Review Board review was obtained from the National Institutes of Health Office of Human Subjects Research because we used existing data without personal identifiers.

### Statistical Analysis

For all primary analyses, follow-up started at age of first DM discharge diagnosis if first diagnosed after 1987, or age in January 1st 1987 otherwise. Patient age was used as the time metric, and late entry into the study was accounted for in all computations. 

To describe survival patterns in patients with DM, we calculated the probability of survival and 95% confidence intervals (CI) using the Kaplan-Meier estimator. A log-rank test was used to compare the survival distributions between categories. 

We estimated the absolute risk of cancer mortality and cancer incidence in DM patients after their first DM diagnosis, accounting for the competing risk caused by non-cancer deaths non-parametrically using (STATA **stcompet**). We also investigated risk and mortality of cancers stratified by gender. Standard errors were computed as described in Marubini & Valsecchi [20]. 

In a secondary analysis, we calculated lifetime risk of developing cancer based on all DM patients (n=1,081). Follow-up started at birth (if born subsequent to January 1, 1958), or at the start of the cancer registry (January 1^st^ 1958) otherwise.

Follow-up ended at age of the event of interest (cancer death for cancer mortality analyses, and age at first primary cancer diagnosis for cancer absolute risk analyses), age at death from non-cancer causes, or age at censoring. Censoring events were emigration or end of study (December 31, 2007). All analyses were performed using STATA12 (StataCorp, College Station, TX, USA)

## Results

The study included 1,081 DM patients who were followed for up to 21 years, contributing 7,735 person-years of follow-up. Approximately half (47.9%) of the patients were male. The median age at DM diagnosis was 46 years (range=0-86), 38.9% of subjects died during follow-up, and 11.5% (n=124) developed cancer (Table S1 shows cancers by anatomic site diagnosed before and after first DM discharge diagnosis) Similar distributions of gender, and age at DM diagnosis were observed when comparing DM patients by source of diagnosis (inpatient *vs.* outpatient). However, more deaths and more cancers occurred in those diagnosed as inpatients, possibly indicating that those hospitalized manifested more severe underlying disease phenotype (Table 1). 

**Table 1:** Characteristics of all myotonic dystrophy patients and by source of first diagnosis.

**Variable**	**All Patients (N=1081)**	**Inpatient Diagnosis (N=718)**	**Outpatient Diagnosis (N=363)**
**N** (**%**)			
**Gender**			
Male	518 (47.9)	339 (47.2)	179 (49.3)
Female	563 (52.1)	379 (52.8)	184 (50.7)
**Age at DM diagnosis (Yrs)**			
Median (range)	46 (0-86)	46.0 (0-86)	44.0 (1-80)
0-33	298 (26.3)	198 (25.9)	100 (27.3)
34-47	305 (26.9)	202 (26.4)	103 (28.1)
48-57	276 (24.4)	183 (23.9)	93 (25.3)
57+	253 (22.4)	182 (23.8)	71 (19.4)
**Calendar year at first DM diagnosis**			
1987-1993	388 (34.3)	388 (50.7)	0
1994-1999	232 (20.5)	232 (30.3)	0
2000-2003	329 (29.1)	101 (13.2)	228 (62.1)
2003-2007	183 (16.2)	44 (5.8)	139 (37.9)
**Developed cancer ever** ^a^	124 (11.5)	99 (13.8)	25 (6.9)
**Age at diagnosis(Yrs**); Median (range)	51 (2-84)	51.5 (2-84)	51 (22-76)

^a^ Either before or after the beginning of prospective follow-up.

### Absolute Risk of Cancer after DM Diagnosis

Among the 1,015 patients without a prior cancer diagnosis at the beginning of follow-up (contributing 7,297 person-years of follow-up), 58 patients developed incident cancers at a median age of 54 years (range=22-84). Female patients developed cancer at a younger age than males (median age=51 years, range=22-74 *vs.* 57, range=43-84, respectively; p=0.02). Cancers of the breast (n=9), endometrium (n=6), and ovary (n=5) were the most frequent neoplasms in women, while lung cancer was the most frequent in men (n=5).

Overall, the absolute risk of cancer after DM diagnosis increased from 1.6% (95%CI=0.4-4.3%), to 5.1% (95% CI=2.8-8.6%) and 8.8% (95% CI=5.7-12.7%) by ages 40, 50 and 60 years, respectively. In analyses stratified by gender, the absolute risk of all cancers combined was significantly higher in females than males (p=0.03): 7.8% (95% CI=3.7-13.9%) and 13.3% (95% CI=7.8-20.2%) *versus* 2.3% (95%CI= 0.7-5.8%) and 4.4% (95%CI=2.0-8.2%) by ages 50 and 60 years, respectively. 

In analyses restricted to female patients, absolute risk of reproductive cancers (endometrium, cervix, ovary, and other female genital organs) was 1.0 % (95%CI=0.09-5.0%), 3.4% (95%CI=1.1-7.9%) and 4.6% (95%CI=1.9-9.4%) compared with risks of non-reproductive cancers (including breast) that were 2% (95%CI=0.4-6%), 4.5% (95%CI=1.7-9.3%), and 9% (95%CI=4.7-14.1%) by ages 40, 50, and 60 years, respectively.

When we evaluated lifetime absolute risk of cancer in all DM patients (i.e., including cancers diagnosed before the first DM diagnosis), estimates were higher, particularly at younger age, than those based on cancers diagnosed after DM diagnosis (0.2% [95% CI=0.02-0.1%] by age 20; 2.6% [95% CI=1.7-3.9%] by age 40; 5.8% [95% CI=4.3-7.6%] by age 50, and 12% [95% CI=9.7-14.6%] by age 60 years). These results however, have to be interpreted with caution because of potential biases relating to DM under-diagnosis or under-reporting.

### Survival Patterns and Absolute Risk of Cancer Mortality after DM Diagnosis

The median age of death among all DM patients was 49.8 years (95% CI=39.8-53.8). DM itself was the most common registered primary cause of death (n=232; 55.1%), followed by cardiovascular disease (n=95; 22.6%; mainly ischemic heart disease, n=39), malignancy (n=42; 10.0%; cancers of the ovary (n=8), brain (n=7), and lung (n=6) were the most common), and respiratory disease (n=13; 3.1%; mainly infections, n=10). Among those for whom DM was the primary cause of death, the most commonly reported secondary causes were: respiratory (n=80, 12 of whom died of respiratory failure), followed by cardiac causes (n=17). The first death among study participants occurred at age 4 months; the first cancer death occurred at age 41 years. 

The absolute risks of cancer mortality after DM diagnosis in all patients increased from 2.3% (95% CI=1.0-4.5%) to 3.9% (95% CI=2.1-6.4%), and 5.9% (95% CI=3.7-8.7%) by ages 50, 60, and 70 years compared with 48.1% (95% CI=35.8-59.3%), 72.7% (95% CI=65.0-78.9%), and 88.6% (95%CI=84.5-91.7%) for non-cancer deaths by the same ages respectively. 

**Figure 2:**
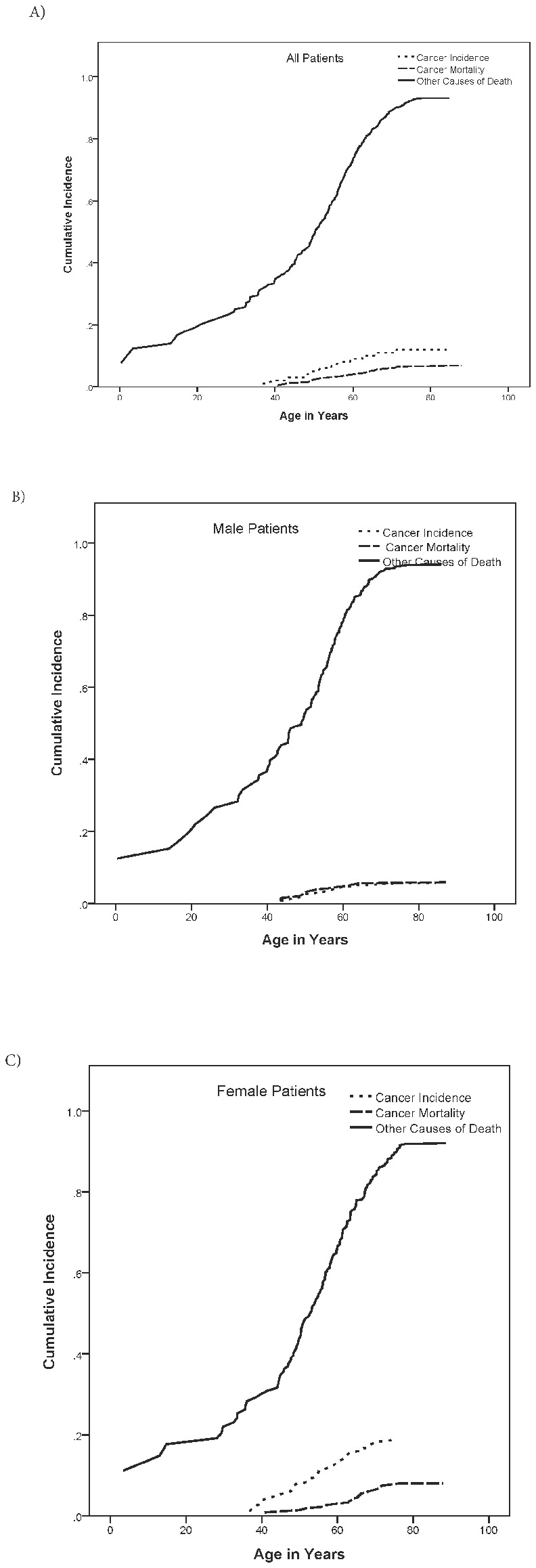
Cumulative incidence curves summarizing cancer burden in patients with myotonic dystrophy. A) Cumulative incidence curves for cancer incidence, cancer mortality and other causes of death in all DM patients; B) Cumulative incidence curves for cancer incidence, cancer mortality and other causes of death in all DM patients in male patients; C) Cumulative incidence curves for cancer incidence, cancer mortality and other causes of death in all DM patients in female patients.

In analyses stratified by gender, male patients had poorer survival overall (63% [95% CI=40-80%]; 45% [95% CI=29-59%]; and 17% [95% CI=12-25%] *vs.* females 70% [95% CI=46-84%]; 55% [95% CI=38-69%]; and 31% [95% CI=21-41%] by ages 40, 50 and 60 years, respectively; log-rank p<0.0001). After accounting for the competing risk of non-cancer deaths, a similar pattern was observed for cancer mortality, particularly in younger patients, although this was not statistically significant (p=0.6). In male patients, the absolute risk of cancer mortality were 3.0% (95% CI=1.0-6.9%), 4.7% (95% CI=2.1-8.8%), and 5.6% (95% CI=2.8-9.9%) *vs.* 1.4% (95% CI=0.2-4.6%), 2.9% (95% CI=1.1-6.3%), and 6.3% (95% CI=3.4-10.4%) in females by age 50, 60, and 70 years, respectively. Figure 2 summarizes the absolute age-specific risks of cancer incidence, cancer mortality, and other causes of death among study participants, overall and by gender. 

## Discussion

We previously quantified the relative risk of cancer among patients with myotonic dystrophy in comparison with the general population [14]. Because of the possible clinical implications of those novel observations, we now present the absolute risk of cancer incidence and cancer mortality after DM diagnosis, while statistically accounting for the high probability of competing non-cancer mortality. Our findings can be used by physicians to communicate cancer risk to their DM patients, and provide a rationale for routinely applying validated, population-appropriate cancer screening strategies to the management of DM patients.

Our study is the first to account for the high competing risk from non-cancer deaths in calculating cancer risk and mortality for DM patients. In the presence of competing mortality, which precludes the occurrence of other outcomes of interest (in this instance, the development of new cancers), it is important to employ a competing risk analysis to avoid overestimating the magnitude of cancer risk [21]. 

Overall, absolute cancer risk after DM diagnosis increased from 1.6% by age 40 years to 5% and 9% by ages 50 and 60 years, respectively. The observed gender difference in the risk of all cancers combined was driven by the high incidence of female cancers, including cancers of the breast and reproductive organs. After excluding cancers of the genital organs and the breast, the absolute risk of the remaining cancers was not statistically different between males and females (p=0.41). Compared with the general population, female DM patients are at high risk of cancers of the endometrium and ovary [14]. The high frequency of breast cancer in female DM patients has been observed previously [14,15,22] (in general, breast cancer is the most common tumors in women) but breast cancer risk was comparable to that of women from the general population [14,15]. Based on that observation, we do not currently view breast cancer as part of the DM phenotype; its presence in this series likely reflects its relatively high incidence in the general population. 

Of interest, and when compared with cancer cumulative incidence of the general Swedish population, the absolute risk of cancer in female (but not male) DM patients exceeded that of the population of the same ages (in females: 14.6% *vs.* 7.7% [23], in males 3.5% *vs.* 4.4% [23] by age 55). The higher death rates among male DM patients *versus* females may contribute to this observation (by 60 years of age, the overall survival probability in male patients is 17% compared with 31% in females). 

Calculating the absolute risk of cancer after DM diagnosis provides clinically useful estimates for physician-patient communication purposes, and minimizes possible biases expected if one used lifetime estimates for this purpose, since there is no way to identify patients who died from cancer prior to their being recognized as having DM. 

In this large population-based study, and in agreement with previous reports, DM patients were at very high risk of premature death. Sweden has one of the highest life expectancies in the world (mean life expectancy = 81.8 years in 2011) [24], while the median age of death for DM patients in this study was 49.8 years, an estimate that is similar to prior reports from the US [11] and the Netherlands [10]. Furthermore, several prior studies have suggested a survival advantage for female DM patients [10,13], an observation that was confirmed in our study overall. Cancer death was the third most common cause of death among DM patients; an observation that had been noted in passing in earlier publications, but which did not attract clinical attention [10–13]. The under-recognition of the cancer mortality burden in DM patients is possibly driven by the significant high competing death attributed to non-cancer causes in those patients. After accounting for the very high competing non-cancer mortality (48%, 73%, and 89% by ages 50, 60, and 70 years), the absolute risk of cancer death in DM patients reached 2%, 4% and 6% by the same ages. While cancer is a distant third on the list of DM-associated causes of death, it nonetheless accounts for 10% of all deaths in DM patients, and it is quite possible that a meaningful fraction of those deaths could be avoided if cancer were consciously considered (by applying validated, population-appropriate screening) in both the day-to-day health care of DM patients, and in the differential diagnosis of new, unexplained medical signs or symptoms. Minimizing diagnostic delay is a worthwhile goal in this context.

The strengths of our study include its population-based design that minimizes selection bias, and the use of registry-based records to verify diagnoses that minimizes recall bias. The study included DM patients identified from both inpatient and outpatient records, improving the external validity and generalizability of our results. The study limitations include the lack of information on known cancer risk factors, genetic subtype of DM disease in affected individuals (i.e., DM1 *versus* DM2), nucleotide repeat length which, at least for DM1, is thought to correlate strongly with disease severity. Previous research [22,25] suggested that there is no association between nucleotide repeat expansion size and cancer risk in DM patients; additional research is needed in this area. It is likely that there are more DM1 cases among patients in the current series since it is both more prevalent in the general population, and it was also characterized genetically before DM2. Due to the small observed numbers of individual site-specific cancers, we were not able to calculate reliable site-specific cancer absolute risks or mortality estimates. Finally, the Swedish database did not contain DM-specific clinical information such as disease severity. 

In conclusion, our study provides the first quantification of cancer burden after DM diagnosis, accounting for the high non-cancer competing mortality associated with this multisystem disease. Importantly, cancer remains a meaningful contributor to DM-related morbidity and, to a lesser extent, mortality. The molecular mechanism underlying the increased cancer risk in DM patients remains to be defined, but several mechanisms have been hypothesized [14,26]. Future research is needed to understand the mechanisms of DM-associated carcinogenesis and to clarify whether nucleotide repeat expansion size is a modifier of this relationship. It is important to incorporate validated population-based cancer screening modalities into the routine clinical care of DM patients, and to evaluate suspicious new symptoms with the possibility of cancer in mind. Early onset active surveillance through detailed medical history and careful physical examination with an eye towards minimizing diagnostic delay is important relative to cancers for which screening has not been proven beneficial, such as ovarian, endometrial and brain cancers. Reevaluation of cancer incidence in DM patients will be necessary as more effective DM treatments are developed, since cancer incidence and its associated mortality burden, are likely to increase as competing risks diminish.

## Supporting Information

Table S1 **Cancer sites before and after start of follow-up.**
(DOCX)
