# Supplementary material for: Quantifying Cancer Absolute Risk and Cancer Mortality in the Presence of Competing Events after a Myotonic Dystrophy Diagnosis
Source: PLoS One. 2013 Nov 13;8(11):e79851. doi: 10.1371/journal.pone.0079851 (PMC3827449; doi:10.1371/journal.pone.0079851)
Supplement: Table S1 — Cancer sites before and after start of follow-up. (DOCX) [file pone.0079851.s001.docx]

Table S1

| Anatomic Site | Before Start of Follow-up | During Follow-up |
| --- | --- | --- |
| **All sites** | **66** | **58** |
| Small Intestine | 2 | 0 |
| Colon | 2 | 4 |
| Rectum and anus | 1 | 3 |
| Pancreas | 0 | 2 |
| Lung | 2 | 5 |
| Melanoma (skin) | 4 | 2 |
| Non-Melanoma (skin) | 2 | 0 |
| Breast | 5 | 9 |
| Endometrium | 4 | 6 |
| Ovary | 7 | 5 |
| Other female genital organs | 1 | 2 |
| Kidney | 2 | 1 |
| Other Urinary organs | 2 | 0 |
| Brain | 16 | 3 |
| Parathyroid | 5 | 2 |
| Non-Hodgkin lymphoma | 3 | 2 |
| Leukemia | 1 | 3 |
| Others | 8^a^ | 9^b^ |

Follow-up starts at first myotonic dystrophy diagnosis between 1987 and 2007

^a^ other sites are unknown, thyroid, penis, bone, uterus (part unspecific), chorioepithelioma, and muscle (1 case each)

^b^ other sites are 3 unknown; and prostate, thyroid, esophagus, liver, cervix, and eye (1 case each)
